# Sensitive multiple myeloma disease monitoring by mass spectrometry

**DOI:** 10.1038/s41408-021-00473-9

**Published:** 2021-04-29

**Authors:** Rasa Santockyte, Chelsea Jin, James Pratt, Ron Ammar, Keyur Desai, Mohan Bolisetty, Prianka Das, Mihaela Popa-McKiver, Oscar Puig

**Affiliations:** 1 Translational Medicine, Bristol Myers Squibb, Lawrence Township, NJ USA; 2 Biometrics and Data Sciences, Bristol Myers Squibb, Lawrence Township, NJ USA; 3 Hematology Clinical Development, Bristol Myers Squibb, Lawrence Township, NJ USA; 4 Present Address: Eli Lilly and Company, 450 East 29th St, New York, NY 10016 USA

**Keywords:** Myeloma, Translational research

Dear Editor,

M-protein detection by serum protein electrophoresis and immunofixation in multiple myeloma is the basis for clinical management. However, low sensitivity, inconsistency of assay results, and therapeutic antibody interference often confound results and negatively impact the accuracy of clinical response assessment. Mass spectrometry has been used as a sensitive method to detect M-protein in myeloma^1^. We applied a high-resolution mass spectrometry (HRMS) assay^2^ to serum samples from the ELOQUENT-3 trial^3^. First, all antibodies are immunoprecipitated from serum, capturing all immunoglobulins and free light chains. Captured intact immunoglobulins are denatured to dissociate the light chains from the heavy chains prior to analysis by mass spectrometry. Both the light and the heavy chain components are detected by mass spectrometry, but only the intensity of light chain is used for relative quantitation. The HRMS intensity for each light chain peak in each sample is normalized (Supplementary information), ensuring that intensity values across samples can be compared. As a result, a single peak with normalized intensity of 0.16 arbitrary units (a.u.) corresponds to 100 mg/L of monoclonal antibody. Normalized peak intensities greater than or equal to 0.16 have signal-to-noise ratios greater than 4, which allows for clear separation of monoclonal light chain peaks from the polyclonal background.

The 112 baseline samples were analyzed, and in 94/112 a single monoclonal light chain peak was identified as a prominent single peak in the mass range of lambda or kappa light chains (22,000–24,400 Da, Supplementary Fig. 1A, B). Eighty-one of those 94 had measurable M-protein as defined by International Myeloma Working Group (IMWG) criteria^4^, while 13/94 had values of SPEP < 5 g/L and UPEP < 0.2 g/24 h, and myeloma disease was determined by sFLC^4^ (Supplementary Table 1). In the 18/112 samples in which more than a single peak was identified, 2/18 had biclonal light chain peaks (Supplementary Fig. 1C), and measurable M-protein according to IMWG criteria. Seven out of 18 had no peak in the lambda/kappa mass range and only displayed multiple peaks in the mass range 25,000–27,000 Da (Supplementary Fig. 1D), with six of them showing measurable M-protein according to IMWG criteria. In the remaining 9/18 samples, HRMS peaks were below 0.16 a.u. normalized intensity and no distinct single monoclonal light chain peak was detected above the polyclonal background (Supplementary Fig. 1E). Eight of them had measurable disease detected only by sFLC and one had measurable disease by sFLC and UPEP assays (Supplementary Table 1).

We investigated whether HRMS could be used to eliminate therapeutic antibody interference. Of the 94 baseline samples for which we could identify a single monoclonal light chain peak, there was 1 sample in which the myeloma peak would overlap with the expected mass from elotuzumab light chain (PID 156, light chain mass of 23,423.4 Da). Next, we investigated whether we could differentiate elotuzumab from myeloma light chains in serum samples from 53 subjects treated with elotuzumab. We observed non-overlapping myeloma and elotuzumab light chain peaks in on-treatment samples analyzed (Supplementary Fig. 2A), with elotuzumab contribution ranging from 0% to 100% of the summed HRMS intensity of the two major peaks. In subject 001, who achieved best overall response (BOR) VGPR, elotuzumab signal was predominant with no detectable monoclonal light chain signal at cycles 11–26 (Supplementary Fig. 2B), and UIFE/SIFE were negative. Of the 11 subjects achieving VGPR, this was the only subject whose clinical assessment seemed impacted by elotuzumab interference.

Next, we investigated whether HRMS could monitor disease when compared with standard methods. For subjects with BOR of PR, MR, SD, or PD, HRMS did not provide any additional clinically meaningful improvement in the detection of disease. In subject 103, who achieved sCR, HRMS did not add additional information since monoclonal light chain was not detectable after cycle 3 (Supplementary Table 2). In contrast, HRMS was very informative in five subjects (PIDs 058, 091, 100, 121, 122) who had measurable disease at baseline and showed reductions in laboratory measurements consistent with CR (bone marrow was not available in all of them to confirm CR; Table 1). In these subjects, HRMS detected monoclonal light chain at all the time points tested, but multiple time points were negative by SPEP/UPEP/SIFE. For example, subject 122 had disease measurable at baseline by SPEP, which became 0 starting at cycle 5, with negative SIFE. Negative SPEP/SIFE persisted through cycle 29 (Fig. 1A and Supplementary Table 3), and at cycle 30 SPEP started to rise (Fig. 1A). Monoclonal light chain was detectable by HRMS at all the time points (Fig. 1A, B), indicating HRMS is more sensitive than standard methods to monitor disease. Furthermore, HRMS could detect a sustained increase in monoclonal light chain starting at cycle 21 (as compared to cycle 30 by SPEP/SIFE). In the subject who achieved CR and measurable disease at baseline was detected by sFLC (PID 058), the sFLC ratio was consistently in normal range in several treatment cycles despite HRMS detecting monoclonal light chain at all times tested (not shown).

**Table 1:** HRMS detects sustained increases in monoclonal light chain before standard methods do.

PID	Arm	BOR (INV)	BOR cycle	Clinical progression method by IMWG	Progression cycle by IMWG	Last cycle measured by data cut-off	Sustained M-protein increase by HRMS	Last cycle measured by HRMS	Difference in cycles, HRMS vs standard method^a^	Notes
001	EPd	VGPR	C7	NA	NA	C26	ND	C26		No documented progression
031	Pd	VGPR	C9	Plasmacytoma	C24	C23	C20	C23	4	C24 was an assessment visit, not a treatment cycle. Progression by SPEP at assessment visit C25
044	EPd	VGPR	C4	UPEP	C9	C9	C6	C9	3	
058	EPd	CR^b^	C25-C26^c^	NA	NA	C27	ND	C39		No documented progression
060	EPd	VGPR	C4	UPEP	C18	C19	C7	C19	11	
084	EPd	VGPR	C9	Plasmacytoma	C18	C17	C15	C17	3	No progression by SPEP/UPEP
087	Pd	VGPR	C2	sFLC	C11	C15	C8	C15	3	No progression by SPEP/UPEP
097	Pd	CR^b^	C11	NA	NA	C24	ND	C36		No documented progression
100	EPd	CR	C13	SPEP	C22	C24	C14	C24	8	
101	Pd	VGPR	C10	sFLC	C18	C19	ND	C19		No progression by SPEP/UPEP
103	EPd	sCR	C5	NA	NA	C24	ND	C28		No documented progression
109	EPd	VGPR	C14	NA	NA	C24	ND	C35		No documented progression
116	EPd	VGPR	C8	NA	NA	C23	C14	C35	9	No documented progression
121	EPd	CR	C4	UPEP	C15	C17	C8	C17	7	
122	Pd	CR^b^	C7	NA	NA	C22	C21	C31		No documented progression
133	EPd	VGPR	C10	NA	NA	C22	C31	C34		No documented progression
151	EPd	VGPR	C6	NA	NA	C20	C15	C25	5	No documented progression

The time point (in treatment cycles) of patient relapse, as well as method used to determine clinical relapse, is shown together with the time point where a sustained increase in monoclonal light chain levels is detected by HRMS.

*PID* patient identification, *BOR* best overall response*, INV* investigator, *IMWG* International Myeloma Working Group, *HRMS* high-resolution mass spectrometry.

^a^As determined by the date of data cut-off.

^b^No bone marrow available to confirm CR.

^c^BOR documented between C25 and C26.

**Fig. 1:**
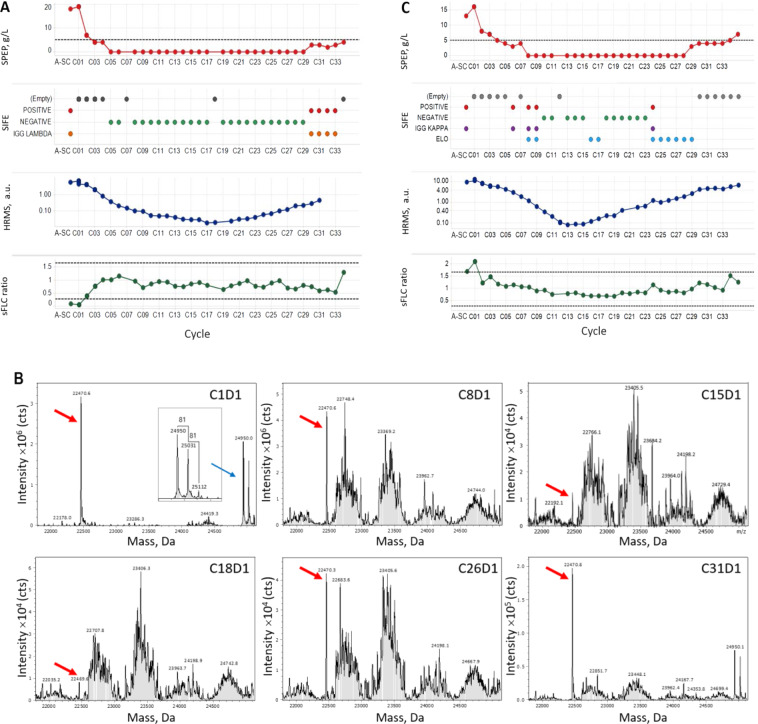
HRMS is more informative and can capture increase in monoclonal light chain earlier than standard methods. **A** SPEP, SIFE, HRMS, and sFLC results for subject 122. **B** HRMS profiles for subject 122 showing detectable monoclonal light chain peak at 22,470.6 ± 1.5 Da in all the time points. The additional peaks in 25,000 mass region represent glycosylated heavy chains at ½ mass based on the difference between the adjacent peaks, which is 81 Da or ½ mass of hexose residue (162 Da). **C** SPEP, SIFE, HRMS, and sFLC results for subject 116.

HRMS was also useful in subjects achieving VGPR. For example, subject 116 had disease measurable at baseline by SPEP. HRMS detected monoclonal light chain in cycles 1–35, but cycles 8–28 were negative by SPEP, and cycles 10–23 negative by SIFE. Cycles 24–29 showed elotuzumab interference by SIFE (Fig. 1C and Supplementary Table 4).

HRMS detected sustained increase in monoclonal light chain starting at cycles where SPEP/SIFE/sFLC were uninformative, therefore, in subjects achieving CR and VGPR (*N* = 16), we investigated at what time points HRMS revealed disease increase as compared with standard clinical assessments (Table 1). In four subjects who had measurable disease by SPEP, HRMS detected sustained increase in monoclonal light chain 3–11 cycles before clinical progression (PID 044, 060, 087, 100). Subject 121 had measurable disease by UPEP and progressed by UPEP at cycle 15; however, HRMS detected sustained increase in monoclonal light chain at cycle 8. In two subjects, clinical progression was due to extramedullary disease (PID 031, 084), and HRMS detected increase 3–4 cycles before SPEP detected increase in M-protein. In three subjects without clinical progression by the date of data cut-off (PID 116, 122, 151), HRMS detected increase 4–9 cycles before the last cycle of clinical assessment. In the remaining five subjects without documented progression (PID 001, 058, 097, 101, 109), HRMS did not detect any increase in monoclonal light chain levels.

Our results show that HRMS can monitor myeloma disease with high sensitivity and specificity, and allows for determination of interference in the assessment of clinical response. These results are in line with prior research^5–8^.

HRMS can monitor decreases in serum monoclonal light chain levels (a surrogate of disease burden) with high sensitivity, and it detects sustained increases at earlier time points, compared to detection of clinical progression by standard methods. In 10 subjects who achieved CR or VGPR, HRMS could detect sustained increases in monoclonal light chain 3–11 cycles earlier than when relapse is determined by clinical assessment. Although limited cases are available, our study shows the value of HRMS in monitoring disease at lower disease burden level. The potential implication of these results is the possibility to identify earlier the patients who begin to relapse, leading to more frequent monitoring or transition onto the next line of therapy. A limitation is its retrospective nature, so defining thresholds to determine increased levels of monoclonal light chain that can prospectively predict clinical relapse will require additional validation studies. Thus, although currently there is no clear threshold of monoclonal light chain increase that would lead to a change in clinical management, our results indicate that this goal is achievable in the near future. Current MRD techniques (Euroflow, clonoSEQ) have increased sensitivity over IFE/sFLC and further research is needed to determine the value of HRMS in MRD assessment.

M-protein half-life is ~2–4 weeks^9^. This prevents using HRMS to monitor short timeframe changes in disease burden due to fast, deep responses. Also, without a baseline sample it is difficult to ensure with certainty that specific peaks detected in treatment samples are responsible for the disease phenotype. In relapsed patient samples, new nascent peaks are detected suggesting they are directly linked to the relapse; however, without a clear understanding of what constitutes healthy polyclonal background, oligoclonal response^10^, and true disease profiles, it is not possible to infer direct causality. In summary, HRMS is a non-invasive, sensitive, and specific method to monitor M-protein in multiple myeloma, shows improved characteristics over current methods, and it has the potential to become a very important tool for disease monitoring.

## Supplementary information

Supplementary information.

Reproducibility checklist.

## Data Availability

https://www.bms.com/researchers-and-partners/independent-research/data-sharing-request-process.html.
