# Supplementary material for: Sensitive multiple myeloma disease monitoring by mass spectrometry
Source: Blood Cancer J. 2021 Apr 29;11(4):78. doi: 10.1038/s41408-021-00473-9 (PMC8084958; doi:10.1038/s41408-021-00473-9)
Supplement: Supplementary file 1 — Supplementary information. [file 41408_2021_473_MOESM1_ESM.pdf]

Supplementary Information for Manuscript "Sensitive multiple myeloma disease monitoring by mass spectrometry"

Rasa Santockyte[1*], Chelsea Jin[2], James Pratt[2], Ron Ammar[1], Keyur Desai[2], Mohan Bolisetty[1], Prianka Das[3], Mihaela Popa-McKiver[3] and Oscar Puig[1,4*]

This PDF file contains

Methods

Supplementary Figures

Supplementary Tables

Methods

Samples and human subjects

Forty-one normal human serum samples were purchased from BioIVT (NY, USA). Twenty-three of these normal samples were pooled and used as calibration samples in each run. Elotuzumab, a humanized IgG1 monoclonal antibody, was added into the calibration samples as a quality control (QC) for each analytical run. Test samples consisted of 1052 serum samples from 112 subjects in ELOQUENT-3[1] (NCT02654132) collected as part of the clinical protocol to analyze M-protein by SPEP for response assessment purposes. One hundred twelve of those 1052 samples were baseline samples collected prior to treatment (at cycle 1 day 1 pre-dose). Bone marrow samples were collected at baseline, at cycle 4 day 1, or at the time of complete response or very good partial response assessment.

All patients provided informed consent for this research. All procedures performed with human samples were in accordance with the ethical standards of the institutional and national research committees, and with the 1964 Helsinki Declaration.

Sample Preparation

Each run had 20 QC samples (four replicates each of elotuzumab added into pooled normal serum at 0, 50, 100, 1000, 3000 mg/L) and 76 test samples. Each test or calibration sample (10 μl) was diluted 40-fold with phosphate-buffered saline (PBS) and 110 µL of diluted sample was used for immunocapture using PhyTip columns (PhyNexus, USA) containing 1:1 mixture of CaptureSelect LC-lambda Hu and CaptureSelect Kappa XL (Thermo Fisher, USA). Automated liquid handling was performed on the Freedom EVO® automation platform equipped with 96 channels (Tecan, USA). PhyTip columns were first washed with PBS and subsequently immunoglobulins were captured from the diluted sera; the columns were then washed with 220 µL of each of wash solutions: wash buffer (10 mM phosphate, 500 mM NaCl and 0.1% zwittergent, pH 7.4), PBS, and water. Immunoglobulins were eluted in elution buffer (12 mM HCl, 100 mM NaCl, pH 2). The eluted samples were reduced immediately by addition of 100 mM buffered Tris(2-carboxyethyl)phosphine solution (Thermo Fisher, USA) to a final concentration of 20 mM followed by incubation at 25°C for 30 min.

Liquid-chromatography mass spectrometry

Chromatography was performed on an Acquity UPLC H-class Bio system (Waters Corporation, USA). Immunoglobulins eluted from PhyTip columns were injected onto a C4 column (Acquity Protein BEH C4 300Å, 1.7 µm, 2.1 mm x 100 mm) at 80°C. Mobile phase A contained 0.1% formic acid (FA) in water and mobile phase B contained 0.1% FA in acetonitrile. A gradient separation was used as follows: 0–1.00 min 5% B; 1.00–2.00 min 10-33% B; 2.00–6.00 min 33–40% B; ramp up to 90% B over 0.01 min; 6.01–8.50 min hold at 90% B; ramp down to 5% B over 0.01 min; 8.51–10.0 min maintain at 5.00% B. The flow rate was set at 0.3 mL/min and the injection volume was 2.0 µL.

The UPLC was connected to a Maxis 4G Q-TOF instrument with a standard Electrospray (ESI) Apollo-source (Bruker Daltonics, Germany). To ensure mass accuracy, the instrument was calibrated before each run by infusing ESI-L low concentration tuning mix (Agilent Technologies, USA). Each 10-min run was segmented as follows: 2 min to

waste, followed by 4 min to source, and the last 4 min to waste. Capillary voltage was set at 4500 V. The nebulizer was set at 1.6 bar. The dry gas was set at 8.5 L/min. The dry temperature was set at 200°C. The ion cooler transfer time was 100 µs, with a prepulse storage of 25 µs. The ion polarity was positive; the rolling average was activated and set at 2. TOF MS scans were acquired from m/z 700-2800 with an acquisition rate of 1.0 Hz. A detailed protocol for sample preparation and liquid chromatography-high-resolution mass spectrometry (LC-HRMS) analysis has been published elsewhere[2].

Mass Spectrometry Data analysis

Mass spectrometry data files were processed using Compass DataAnalysis 5.2 software, with analyte retention time of 4.25 ± 0.75min, peak spectrum extraction window between 700 and 2800 m/z, and maximum entropy deconvolution range from 20 000 to 28 000 Da. To remove possible batch effects, the geometric mean of the four 1000 mg/L QC sample replicates within each run was used to normalize peak intensities in the samples in that run, and when possible, all samples from the same subject were processed in the same batch. Single peak analysis was performed by identifying the monoclonal light chain (derived from intact monoclonal M-protein or from monoclonal free light chain) at baseline as the highest peak in the analyzed mass range and following the same peak mass (± 1.5 Da) in subsequent time points. The computer code for single peak analysis was implemented in Python (the code is available upon request). To determine at which cycle sustained increases in monoclonal light chain start for each subject, we inspected HRMS measurements in log10 scale and visually identified unambiguous inflection points, indicating uptick in monoclonal light chain (for example cycle 21 in HRMS panel in Fig. 1A, or cycle 14 in Fig. 1C). If no sustained increase was clearly detected, PID was assigned an ND (increase not detected).

Standard clinical response measurements

Serum and urine M-protein, and serum free light chain were measured as part of standard clinical response assessments following the International Myeloma Working Group guidelines[3] and the data have been published

elsewhere[1]. The data cut-off for clinical assessments was November 29th, 2018. Best Overall Response (BOR), as assessed by the study investigators, was used as a clinical response parameter.

References

1 Dimopoulos, M. A. *et al.* Elotuzumab plus Pomalidomide and Dexamethasone for Multiple Myeloma. *N Engl J Med* **379**, 1811-1822, doi:10.1056/NEJMoa1805762 (2018).

2 Santockyte, R. *et al.* High-Throughput Therapeutic Antibody Interference-Free High-Resolution Mass Spectrometry Assay for Monitoring M-Proteins in Multiple Myeloma. *Analytical Chemistry* **93**, 834-842, doi:10.1021/acs.analchem.0c03357 (2021).

3 Rajkumar, S. V. *et al.* International Myeloma Working Group updated criteria for the diagnosis of multiple myeloma. *Lancet Oncol* **15**, e538-548, doi:10.1016/S1470-2045(14)70442-5 (2014).

Supplementary Figures

Supplementary Figure 1

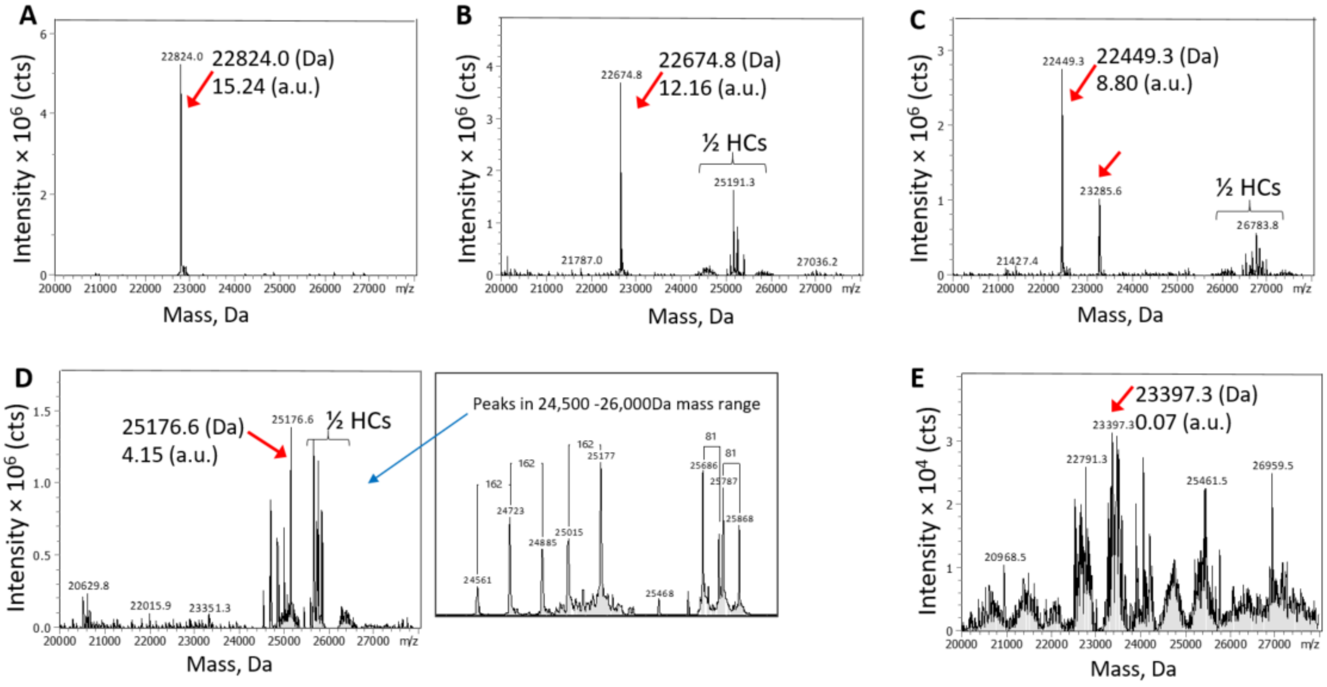

Supplementary Fig. 1 HRMS profiles. (A) a profile showing a single light chain peak at mass 22,824.0; (B) a profile showing glycosylated heavy chain at mass 25,191.3 in addition to a single light chain peak at mass 22,674.8; (C) a profile showing two light chain peaks, at masses 22,449.3 and 23,285.6, and glycosylated heavy chain at 26,783.8; (D) a profile showing glycosylated light and heavy chains outside the known regions for lambda and kappa;* (E) a profile of a sample where no distinct monoclonal light chain peak was identified. Compare Y axis scale to the one in Supplemental Fig 1A-D.

* Glycosylated light chains can be distinguished from glycosylated heavy chains based on the mass difference between the adjacent peaks. Light chains are spaced one sugar unit apart (in this case 162 Da, which is equal to one hexose residue mass), while heavy chains are spaced half sugar units apart (in this case 81 Da, which is half mass of hexose residue).

Supplementary Figure 2A

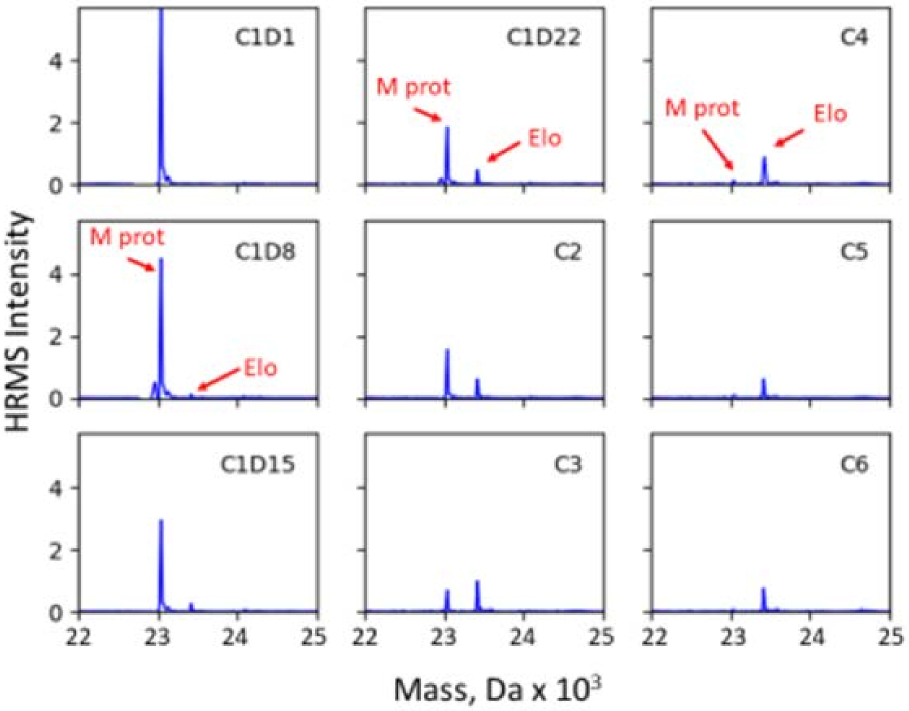

Supplementary Fig. 2A Detection of antibody interference in several time points for subject 121. M-protein, as identified in the baseline sample, is detectable in all time points. Elotuzumab peak is detectable starting at cycle 1 day 8.

Supplementary Figure 2B

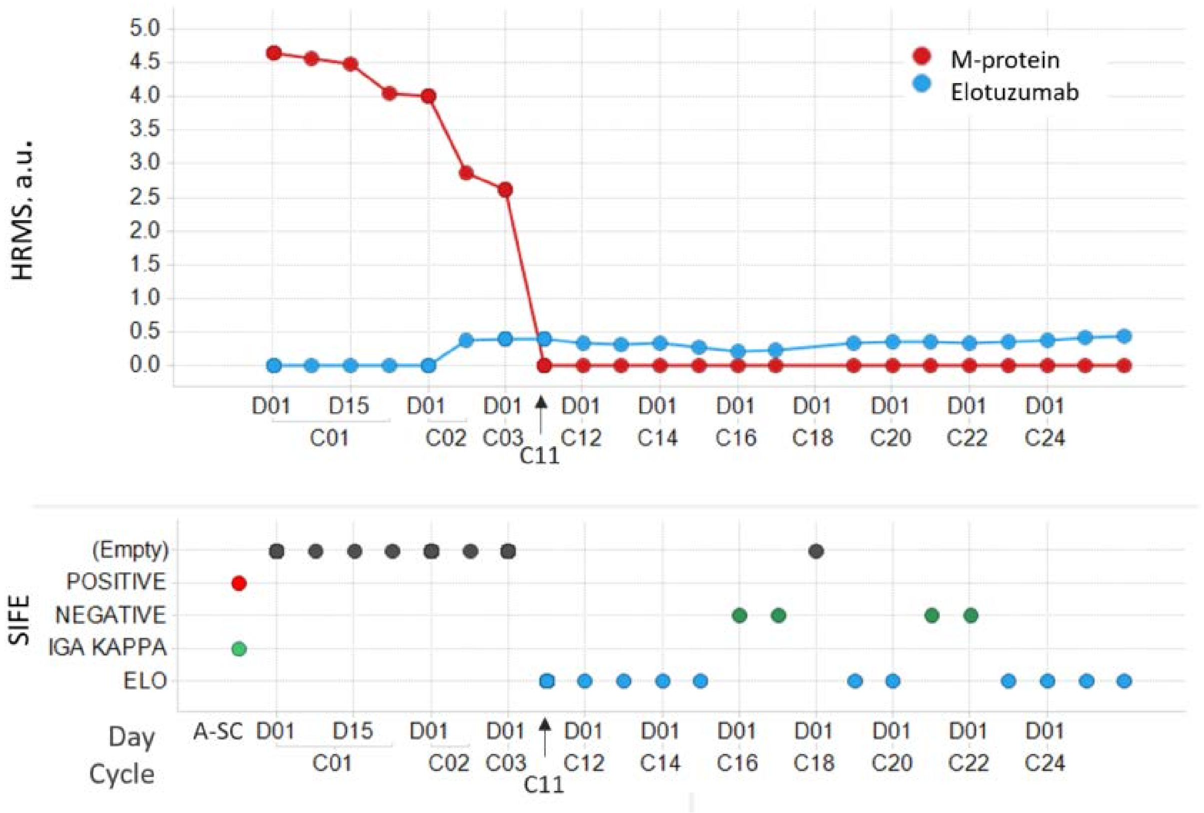

Supplementary Fig. 2B Graph displaying elotuzumab and M-protein normalized intensities for subject 001.

Elotuzumab does not interfere with M-protein measurement by HRMS.

Supplementary Tables

Supplementary Table 1. M-protein peak position for all baseline samples in ELOQUENT-3 as detected by HRMS (C1D1), and SPEP/UPEP/sFLC (screening) values.

PID	Arm	Mass, Da	Peak intensity, cts	Normalized intensity, a.u.	Measurable M-protein according to IMWG	SPEP, g/L	UPEP, g/24h	Kappa, mg/L	Lambda, mg/L	sFLC ratio
154	Pd	23484.5	7089283	23.16	YES	33	0.04	180.00	1.92	93.75
073	E-Pd	23330.6	6586683	20.11	YES	35	0.00	73.10	1.05	69.62
131	E-Pd	23264.3	5706033	19.96	YES	32	0.01	147.00	1.17	125.64
083	Pd	22739.4	5763654	19.84	YES	37	0.97	7.16	662.00	0.01
094	E-Pd	23361.2	6562329	19.80	YES	26	0.20	514.00	10.20	50.39
085	E-Pd	23078.3	5563505	19.46	YES	28	0.02	7.49	369.00	0.02
138	Pd	22822.8	5815088	18.75	YES	27	0.02	1.98	15.90	0.12
156	E-Pd	23423.4	5400023	18.59	YES	20	0.07	127.00	1.39	91.37
142	E-Pd	22551.8	5515949	18.54	YES	43	0.02	3.15	322.00	0.01
037	Pd	23266.7	5507149	18.51	YES	54	0.00	46.70	1.62	28.83
112	Pd	22724.8	5613113	18.10	YES	5	0.33	6.84	421.00	0.02
113	Pd	23532.6	5613113	18.10	YES	36	0.57	685.00	1.90	360.53
137	Pd	23581.7	5156158	17.33	YES	11	0.24	533.00	9.97	53.46
139	Pd	23485.6	5132510	17.25	YES	28	0.08	1600.00	9.01	177.58
096	E-Pd	23181.1	4999017	17.21	YES	42	0.40	339.00	13.90	24.39
132	E-Pd	23059.2	5250375	16.93	YES	42	0.07	524.00	6.02	87.04
040	Pd	23318.4	4827978	16.89	YES	17	0.24	990.00	2.57	385.21
084	E-Pd	23419.5	5510346	16.82	YES	24	0.03	772.00	5.50	140.36
003	Pd	23625.6	5521255	16.66	YES	28	0.03	23.50	5.49	4.28
144	E-Pd	23475.8	5158176	16.29	YES	22	0.00	30.80	0.50	61.60
023	Pd	23169.3	4530542	15.85	YES	13	0.00	42.60	1.80	23.67
145	Pd	23869.0	4569468	15.73	YES	38	1.07	154.00	5.26	29.28
133	E-Pd	23325.6	4622929	15.54	YES	17	0.00	483.00	8.98	53.79
143	E-Pd	22664.4	4422795	15.36	YES	46	0.01	3.25	5.65	0.58

PID	Arm	Mass, Da	Peak intensity, cts	Normalized intensity, a.u.	Measurable M-protein according to IMWG	SPEP, g/L	UPEP, g/24h	Kappa, mg/L	Lambda, mg/L	sFLC ratio
007	Pd	23042.0	4372711	15.30	YES	31	0.21	2.55	370.00	0.01
020	Pd	22824.0	4991048	15.24	YES	38	0.42	6.05	550.00	0.01
092	E-Pd	23417.5	4713174	15.20	YES	49	0.01	14.70	1.51	9.74
072	E-Pd	23315.6	4831660	14.75	YES	18	0.00	56.80	9.18	6.19
079	E-Pd	23476.6	4673880	14.10	YES	12	0.02	177.00	15.20	11.64
116	E-Pd	23757.9	4213362	13.82	YES	13	0.00	19.00	11.30	1.68
032	E-Pd	22056.5	4249556	12.98	YES	23	0.73	2.49	5490.00	0.00
005	Pd	23256.7	3837780	12.95	YES	41	0.15	754.00	7.03	107.25
108	E-Pd	23315.8	3680189	12.78	YES	14	0.02	18.70	21.30	0.88
049	E-Pd	23464.2	3785807	12.72	YES	31	0.03	82.50	2.25	36.67
152	Pd	23350.9	3916891	12.37	YES	17	0.00	45.90	5.31	8.64
067	Pd	22976.7	3741047	12.22	YES	14	0.36	10.50	190.00	0.06
100	E-Pd	23669.3	3784258	12.20	YES	27	0.05	27.90	8.61	3.24
136	E-Pd	22674.8	3531956	12.16	YES	17	0.00	1.96	120.00	0.02
044	E-Pd	23436.3	3450095	12.07	YES	19	0.32	2060.00	1.57	1312.10
129	Pd	22354.0	3465956	12.04	YES	16	0.74	8.07	1190.00	0.01
030	E-Pd	23547.7	3781915	11.95	YES	8	0.02	59.50	2.96	20.10
064	Pd	23100.5	3532736	11.87	YES	14	0.00	26.80	22.90	1.17
099	Pd	22536.3	3522234	11.84	YES	13	2.56	6.70	1340.00	0.01
021	Pd	23674.9	3328959	11.65	YES	29	0.00	13.80	2.49	5.54
038	Pd	23626.4	3662419	11.57	YES	20	0.51	1930.00	1.15	1678.26
135	E-Pd	24323.8	3188041	11.07	YES	29	0.37	7490.00	1.43	5237.76
105	Pd	22698.0	3255935	10.68	YES	35	0.00	1.32	9.64	0.14
068	Pd	22683.6	3077292	10.59	YES	13	0.32	29.60	158.00	0.19
060	E-Pd	23451.5	3063269	10.54	YES	24	0.79	120.00	2.28	52.63
025	E-Pd	23271.3	3335810	10.54	YES	20	0.05	21.10	3.76	5.61
056	E-Pd	23501.3	3414802	10.43	YES	33	0.31	658.00	23.90	27.53
086	E-Pd	23489.9	3654663	10.24	YES	4	1.42	725.00	0.50	1450.00
110	E-Pd	23865.8	2892047	10.05	YES	1	2.87	1240.00	1.37	905.11

PID	Arm	Mass, Da	Peak intensity, cts	Normalized intensity, a.u.	Measurable M-protein according to IMWG	SPEP, g/L	UPEP, g/24h	Kappa, mg/L	Lambda, mg/L	sFLC ratio
012	E-Pd	23136.4	2776794	9.71	YES	35	0.03	12.20	218.00	0.06
128	Pd	23556.6	2761863	9.28	YES	16	0.11	258.00	6.41	40.25
095	E-Pd	23635.8	2680806	9.23	YES	6	0.52	10.30	7170.00	0.00
146	Pd	22481.8	2710836	8.89	YES	12	0.00	6.65	16.20	0.41
147	E-Pd	23478.8	2532606	8.17	YES	14	0.08	45.50	12.40	3.67
036	Pd	23562.8	2317044	7.82	YES	6	0.20	157.00	26.50	5.92
093	Pd	22742.1	2221606	7.47	YES	18	0.05	12.90	866.00	0.01
052	Pd	22737.0	2093061	7.20	YES	2	0.82	11.00	3940.00	0.00
062	E-Pd	23367.5	2199418	7.19	YES	6	0.00	15.70	11.00	1.43
031	Pd	23492.6	2367774	7.14	YES	18	0.00	17.20	2.61	6.59
150	E-Pd	24048.0	2503876	7.08	YES	11	0.67	38.30	6.28	6.10
122	Pd	22470.5	2121578	6.84	YES	18	0.01	21.00	274.00	0.08
027	Pd	24069.7	2088758	6.74	NO	1	0.14	2770.00	0.50	5540.00
070	Pd	22642.5	1940550	6.36	NO	3	0.00	7.83	241.00	0.03
091	E-Pd	23355.5	2105109	6.35	YES	10	0.08	65.10	9.00	7.23
043	E-Pd	23773.1	2137641	5.93	YES	11	0.00	6.49	1.69	3.84
121	E-Pd	23040.2	1796741	5.71	YES	2	0.37	1.45	1910.00	0.00
001	E-Pd	23433.7	1616357	4.88	YES	18	0.00	768.00	5.61	136.90
047	Pd	23439.9	1466028	4.48	YES	1	4.33	4120.00	8.02	513.72
010	E-Pd	23885.4	1310924	4.42	YES	1	6.76	3570.00	1.38	2586.96
097	Pd	23173.7	1069824	3.72	YES	11	0.14	398.00	29.80	13.36
015	E-Pd	23550.6	1015351	3.42	YES	1	0.79	3800.00	13.90	273.38
054	Pd	23285.8	1023786	3.35	YES	4	0.61	400.00	2.51	159.36
019	E-Pd	23438.9	886487	2.99	NO	2	0.00	1300.00	2.41	539.42
081	E-Pd	23481.1	860851	2.72	YES	1	0.91	3300.00	6.73	490.34
109	E-Pd	22929.0	694398	2.24	YES	21	0.01	8.76	7.22	1.21
041	Pd	22402.1	597483	1.95	YES	0	0.78	1.47	1040.00	0.00
115	Pd	22917.7	477783	1.54	NO	0	0.18	8.58	662.00	0.01
087	Pd	22629.5	422318	1.33	YES	0	1.02	8.16	732.00	0.01

PID	Arm	Mass, Da	Peak intensity, cts	Normalized intensity, a.u.	Measurable M-protein according to IMWG	SPEP, g/L	UPEP, g/24h	Kappa, mg/L	Lambda, mg/L	sFLC ratio
157	Pd	22740.4	383775	1.32	NO	0	0.13	2.72	558.00	0.00
042	E-Pd	22711.2	306497	1.00	YES	1	1.89	8.68	697.00	0.01
104	E-Pd	22780.4	269599	0.91	YES	0	1.06	1.96	671.00	0.00
090	Pd	22720.7	216791	0.68	NO	0	0.00	1.97	632.00	0.00
069	Pd	22473.0	193836	0.64	NO	0	0.13	9.86	431.00	0.02
029	Pd	23292.9	174152	0.61	NO	0	0.01	1030.00	7.20	143.06
008	Pd	23439.4	167507	0.55	NO	0	0.17	599.00	6.92	86.56
035	E-Pd	22599.5	166662.5	0.55	NO	2	0.10	0.90	167.00	0.01
058	E-Pd	22747.0	175423	0.54	NO	0	0.01	2.49	445.00	0.01
004	E-Pd	23021.3	112206	0.38	NO	0	0.02	1.29	203.00	0.01
088	Pd	23447.2	66789	0.23	NO	0	0.00	457.00	9.06	50.44
002	Pd	25239.8	4366515	13.79	YES	3	4.24	0.90	12400.00	0.00
155	E-Pd	26435.5	2924023	10.16	YES	35	0.01	2340.00	1.04	2250.00
024	Pd	22449.3	2608102	8.80	YES	44	2.05	9.28	1460.00	0.01
119	E-Pd	25973.7	2464875	8.08	YES	15	0.02	639.00	13.80	46.30
089	Pd	22347.3	2032697	6.13	YES	9	0.00	0.90	10.30	0.09
046	E-Pd	22598.4	1958319	5.91	YES	19	0.01	15.60	152.00	0.10
013	E-Pd	25380.5	1521198	4.97	YES	24	0.05	138.00	7.75	17.81
151	Pd	25176.6	1194542	4.15	YES	53	0.00	126.00	5.59	22.54
149	E-Pd	25872.1	777120	2.47	YES	16	0.03	20.10	9.55	2.10
017	Pd	25906.6	136039	0.41	NO	0	0.06	1930.00	2.25	857.78
050	Pd	26156.7	47764	0.15	YES	0	0.89	1240.00	1.15	1078.26
123	Pd	25889.1	36198	0.13	NO	0	0.02	1280.00	5.55	230.63
065	E-Pd	23320.7	34140	0.12	NO	0	0.00	365.00	0.53	688.68
101	Pd	23524.2	37689	0.12	NO	0	0.10	2710.00	1.20	2258.33
103	E-Pd	23512.2	31091	0.10	NO	0	0.01	149.00	8.37	17.80
071	E-Pd	23527.5	25463	0.09	NO	0	0.02	396.00	14.20	27.89
127	E-Pd	23074.3	26767	0.08	NO	0	0.00	913.00	1.91	478.01
016	E-Pd	24082.3	23278	0.07	NO	0	0.02	85.50	19.00	4.50
076	Pd	26001.4	16610	0.06	NO	0	0.00	426.00	15.00	28.40

Supplementary Table 2. SPEP, SIFE, sFLC and HRMS M-protein measurements in subject 103.

PID	Cycle	Day	M-protein mass, Da	M-protein intensity, a.u.	Elo mass, Da	Elo, a.u.	% M-protein	% Elo	SPEP, g/L	SIFE	Class (SIFE)	UIFE	Class (UIFE)	sFLC ratio
103	Screen	NA							0	POSITIVE	KAPPA LC	POSITIVE	KAPPA LC	
103	C01	D01	23512.2	0.17	0.0	0.00	100.00	0.00	0	POSITIVE	KAPPA LC	NEGATIVE		47.34
103	C01	D08	23512.3	0.16	23422.6	0.22	42.06	57.94						
103	C01	D15	23512.2	0.14	23422.7	0.29	31.86	68.14						
103	C01	D22	23512.2	0.12	23422.4	0.16	42.15	57.85						
103	C02	D01	23512.1	0.11	23422.7	0.22	33.37	66.63	2	ELO		NEGATIVE		1.35
103	C03	D01	23513.4	0.05	23422.8	0.45	10.75	89.25	2	ELO		NEGATIVE		0.75
103	C04	D01		0.00	23424.1	0.36	0.00	100.00	2	ELO		NEGATIVE		1.11
103	C06	D01		0.00	23424.2	0.51	0.00	100.00	1	ELO		NEGATIVE		0.57
103	C07	D01		0.00	23424.1	0.53	0.00	100.00	1	ELO		NEGATIVE		1.04
103	C08	D01		0.00	23424.2	0.55	0.00	100.00	1	ELO		NEGATIVE		1.46
103	C09	D01		0.00	23424.2	0.64	0.00	100.00	2	ELO		NEGATIVE		1.13
103	C10	D01		0.00	23424.1	0.67	0.00	100.00	1	ELO		NEGATIVE		1.24
103	C11	D01		0.00	23424.3	0.61	0.00	100.00	1	ELO		NEGATIVE		0.41
103	C12	D01	23513.0	0.02	23424.3	0.56	3.21	96.79	1	ELO		NEGATIVE		0.25
103	C13	D01	23513.5	0.02	23424.2	0.59	3.56	96.44	1	ELO		NEGATIVE		0.24
103	C14	D01		0.00	23424.1	0.52	0.00	100.00	0	ELO		NEGATIVE		0.62
103	C15	D01		0.00	23424.2	0.51	0.00	100.00	0	ELO		NEGATIVE		0.26
103	C16	D01		0.00	23424.2	0.54	0.00	100.00	1	ELO		NEGATIVE		1.00
103	C17	D01		0.00	23424.2	0.53	0.00	100.00	1	ELO		NEGATIVE		0.27
103	C18	D01		0.00	23424.3	0.59	0.00	100.00	1	ELO		NEGATIVE		0.22
103	C19	D01		0.00	23424.1	0.64	0.00	100.00	1	ELO		NEGATIVE		0.16
103	C20	D01		0.00	23424.1	0.70	0.00	100.00	1	ELO		NEGATIVE		2.24
103	C21	D01		0.00	23424.1	0.78	0.00	100.00	1	ELO		NEGATIVE		0.53
103	C22	D01		0.00	23424.1	0.68	0.00	100.00	1	ELO		NEGATIVE		0.50
103	C23	D01		0.00	23424.1	0.69	0.00	100.00	1	ELO		NEGATIVE		0.38
103	C24	D01		0.00	23424.1	0.68	0.00	100.00	1	ELO		NEGATIVE		0.66
103	C25	D01		0.00	23424.1	0.73	0.00	100.00	1	ELO		NEGATIVE		0.61

PID	Cycle	Day	M-protein mass, Da	M-protein intensity, a.u.	Elo mass, Da	Elo, a.u.	% M-protein	% Elo	SPEP, g/L	SIFE	Class (SIFE)	UIFE	Class (UIFE)	sFLC ratio
103	C26	D01		0.00	23424.1	0.51	0.00	100.00	1	POSITIVE	IGG KAPPA	NEGATIVE		0.81
103	C27	D01		0.00	23424.2	0.69	0.00	100.00	1	ELO		NEGATIVE		0.22
103	C28	D01		0.00	23424.2	0.65	0.00	100.00	1	ELO		NEGATIVE		0.29
103	EOT	NA		0.00	23424.3	0.35	0.00	100.00	0	ELO		NEGATIVE		0.83
103	CR	NA		0.00	23422.8	0.44	0.00	100.00						
103	Unsched	NA		0.00	23423.8	0.23	0.00	100.00	1	POSITIVE	LAMBDA LC	NEGATIVE		0.34

Supplementary Table 3. SPEP, SIFE, sFLC and HRMS M-protein measurements in subject 122.

PID	Cycle	Day	M-protein mass, Da	M-protein intensity, a.u.	Elo mass, Da	Elo, a.u.	% M-protein	% Elo	SPEP, g/L	SIFE	Class (SIFE)	UIFE	Class (UIFE)	sFLC ratio
122	Screen		22471.0	6.15	23422.2	0.03	99.53	0.47	18	POSITIVE	IGG LAMBDA	POSITIVE	IGG LAMBDA	0.08
122	C01	D01	22470.5	6.39	23421.9	0.03	99.60	0.40	19					0.05
122	C01	D08	22470.5	6.49	23422.6	0.03	99.58	0.42						
122	C01	D15	22470.5	5.20	23422.0	0.04	99.33	0.67						
122	C01	D22	22470.5	4.50	23421.9	0.05	99.01	0.99						
122	C02	D01	22470.5	4.29	23421.4	0.04	99.11	0.89	7					0.39
122	C03	D01	22470.7	2.07	23421.3	0.03	98.40	1.60	4					0.76
122	C04	D01	22470.9	0.85	0.0	0.00	100.00	0.00	4					1.01
122	C05	D01	22470.8	0.39	0.0	0.00	100.00	0.00	0	NEGATIVE		NEGATIVE		1.01
122	C06	D01	22470.7	0.21	0.0	0.00	100.00	0.00	0	NEGATIVE		NEGATIVE		1.15
122	C07	D01	22470.4	0.15	23422.5	0.04	80.08	19.92						
122	C08	D01	22470.6	0.10	0.0	0.00	100.00	0.00	0	NEGATIVE		NEGATIVE		0.95
122	C09	D01	22470.7	0.09	0.0	0.00	100.00	0.00	0	NEGATIVE		NEGATIVE		0.70
122	C10	D01	22470.3	0.05	0.0	0.00	100.00	0.00	0	NEGATIVE		NEGATIVE		0.85
122	C11	D01	22470.8	0.05	0.0	0.00	100.00	0.00	0	NEGATIVE		NEGATIVE		0.93
122	C12	D01	22470.3	0.05	0.0	0.00	100.00	0.00	0	NEGATIVE		NEGATIVE		0.92
122	C13	D01	22470.4	0.04	0.0	0.00	100.00	0.00	0	NEGATIVE		NEGATIVE		0.77
122	C14	D01	22470.6	0.03	0.0	0.00	100.00	0.00	0	NEGATIVE		NEGATIVE		0.75
122	C15	D01	22470.0	0.03	23422.0	0.06	32.57	67.43	0	NEGATIVE		NEGATIVE		0.85
122	C16	D01	22469.6	0.03	0.0	0.00	100.00	0.00	0	NEGATIVE		NEGATIVE		0.91
122	C17	D01	22471.0	0.02	0.0	0.00	100.00	0.00	0	NEGATIVE		NEGATIVE		0.80
122	C19	D01	22469.6	0.02	0.0	0.00	100.00	0.00	0	NEGATIVE		NEGATIVE		0.64
122	C20	D01	22470.0	0.02	0.0	0.00	100.00	0.00	0	NEGATIVE		NEGATIVE		0.78
122	C21	D01	22469.1	0.03	0.0	0.00	100.00	0.00	0	NEGATIVE		NEGATIVE		0.86
122	C22	D01	22470.0	0.03	0.0	0.00	100.00	0.00	0	NEGATIVE		NEGATIVE		0.97
122	C23	D01	22470.3	0.04	0.0	0.00	100.00	0.00	0	NEGATIVE		NEGATIVE		0.77
122	C24	D01	22470.1	0.06	0.0	0.00	100.00	0.00	0	NEGATIVE		NEGATIVE		0.73
122	C25	D01	22470.8	0.07	0.0	0.00	100.00	0.00	0	NEGATIVE		NEGATIVE		0.88

PID	Cycle	Day	M-protein mass, Da	M-protein intensity, a.u.	Elo mass, Da	Elo, a.u.	% M-protein	% Elo	SPEP, g/L	SIFE	Class (SIFE)	UIFE	Class (UIFE)	sFLC ratio
122	C26	D01	22470.3	0.10	0.0	0.00	100.00	0.00	0	NEGATIVE		NEGATIVE		0.98
122	C27	D01	22470.7	0.12	0.0	0.00	100.00	0.00	0	NEGATIVE		NEGATIVE		0.69
122	C28	D01	22470.7	0.20	0.0	0.00	100.00	0.00	3	NEGATIVE		NEGATIVE		0.65
122	C29	D01	22470.7	0.22	0.0	0.00	100.00	0.00	3	NEGATIVE		NEGATIVE		0.80
122	C30	D01	22470.7	0.28	0.0	0.00	100.00	0.00	2	POSITIVE	IGG LAMBDA	NEGATIVE		0.74
122	C31	D01	22470.8	0.46	0.0	0.00	100.00	0.00	3	POSITIVE	IGG LAMBDA	NEGATIVE		0.58
122	C32	D01							4	POSITIVE	IGG LAMBDA	NEGATIVE		0.61
122	C33	D01							0	POSITIVE	IGG LAMBDA	NEGATIVE		0.53
122	CR		22470.5	0.16	0.0	0.00	100.00	0.00						

Supplementary Table 4. SPEP, SIFE, sFLC and HRMS M-protein measurements in subject 116.

PID	Cycle	Day	M-protein mass, Da	M-protein intensity, a.u.	Elo mass, Da	Elo, a.u.	% M-protein	% Elo	SPEP, g/L	SIFE	Class (SIFE)	UIFE	Class (UIFE)	sFLC ratio
116	Screen		23758.8	9.75	0.0	0.00	100.00	0.00	13	POSITIVE	IGG KAPPA	POSITIVE	IGG KAPPA	1.68
116	C01	D01	23757.9	13.21	0.0	0.00	100.00	0.00	16					2.09
116	C01	D08	23758.0	12.72	23422.9	0.06	99.49	0.51						
116	C01	D15	23758.0	10.74	23422.8	0.12	98.87	1.13						
116	C01	D22	23758.0	9.62	23423.0	0.18	98.18	1.82						
116	C02	D01	23758.1	8.63	23422.9	0.22	97.56	2.44	8					1.21
116	C03	D01	23758.1	6.47	23422.8	0.41	94.07	5.93	7					1.46
116	C04	D01	23759.0	5.84	23423.8	0.32	94.86	5.14	5					1.16
116	C05	D01	23759.1	4.31	23423.7	0.29	93.78	6.22	4					1.07
116	C06	D01	23759.2	3.01	23424.0	0.29	91.10	8.90	3	POSITIVE	IGG KAPPA	NEGATIVE		1.14
116	C07	D01	23759.3	1.95	23423.8	0.32	86.05	13.95	4					1.06
116	C08	D01	23759.4	1.17	23423.8	0.34	77.23	22.77	0	ELO	IGG KAPPA	NEGATIVE		1.04
116	C09	D01	23759.5	0.62	23423.9	0.32	65.94	34.06	0	ELO	IGG KAPPA	NEGATIVE		0.88
116	C10	D01	23759.5	0.34	23423.7	0.31	52.32	47.68	0	NEGATIVE		NEGATIVE		0.91
116	C11	D01	23759.8	0.20	23423.8	0.28	41.79	58.21	0	NEGATIVE		NEGATIVE		0.74
116	C13	D01	23759.6	0.08	23423.5	0.26	23.44	76.56	0	NEGATIVE		NEGATIVE		0.78
116	C14	D01	23759.2	0.09	23423.4	0.29	22.69	77.31	0	NEGATIVE		NEGATIVE		0.81
116	C15	D01	23759.4	0.09	23423.5	0.31	22.10	77.90	0	NEGATIVE		NEGATIVE		0.71
116	C16	D01	23759.6	0.12	23423.6	0.34	26.08	73.92	0	ELO		NEGATIVE		0.68
116	C17	D01	23760.0	0.16	23423.7	0.31	33.29	66.71	0	ELO		NEGATIVE		0.69
116	C18	D01	23759.8	0.22	23423.7	0.26	46.35	53.65	0	NEGATIVE		NEGATIVE		0.69
116	C19	D01	23759.3	0.22	23423.5	0.21	51.74	48.26	0	NEGATIVE		NEGATIVE		0.67
116	C20	D01	23759.3	0.41	23423.8	0.26	61.62	38.38	0	NEGATIVE		NEGATIVE		0.80
116	C21	D01							0	NEGATIVE		NEGATIVE		0.77
116	C22	D01	23759.4	0.57	23424.0	0.25	69.33	30.67	0	NEGATIVE		NEGATIVE		0.84
116	C23	D01	23759.3	0.65	23423.7	0.15	80.85	19.15	0	NEGATIVE		NEGATIVE		0.8
116	C24	D01	23759.2	1.23	0.0	0.00	100.00	0.00	0	POSITIVE, ELO ELO	IGG KAPPA	NEGATIVE		1.13

116	C25	D01	23759.2	1.00	23423.3	0.15	86.98	13.02	0	ELO		NEGATIVE		0.92

PID	Cycle	Day	M-protein mass, Da	M-protein intensity, a.u.	Elo mass, Da	Elo, a.u.	% M-protein	% Elo	SPEP, g/L	SIFE	Class (SIFE)	UIFE	Class (UIFE)	sFLC ratio
116	C26	D01	23759.3	1.22	23423.7	0.25	83.17	16.83	0	ELO		NEGATIVE		0.84
116	C27	D01	23759.3	1.54	23423.8	0.27	84.89	15.11	0	ELO		POSITIVE	KAPPA LC	0.87
116	C28	D01	23759.3	1.94	23423.7	0.27	87.77	12.23	0	ELO		POSITIVE	KAPPA LC	0.80
116	C29	D01	23759.2	2.64	23423.8	0.30	89.76	10.24	3	ELO		NEGATIVE		0.97
116	C30	D01	23759.2	4.29	23423.8	0.29	93.74	6.26	4					1.21
116	C31	D01	23759.1	4.72	23423.9	0.27	94.69	5.31	4					1.15
116	C32	D01	23759.2	4.82	23423.8	0.23	95.38	4.62	4					1.02
116	C33	D01	23759.2	4.58	23423.7	0.18	96.16	3.84	4					0.92
116	C34	D01	23759.1	5.90	23424.0	0.17	97.16	2.84	5					1.51
116	C35	D01	23759.1	6.96	23423.9	0.17	97.65	2.35	7					1.25
116	CR	CR	23759.7	0.10	23423.8	0.20	33.71	66.29						
